# Using personas and the ADKAR framework to evaluate a network designed to facilitate sustained change toward active learning in the undergraduate classroom

**DOI:** 10.1007/s44217-022-00023-w

**Published:** 2022-12-27

**Authors:** Amy J. Prunuske, Heather J. Evans-Anderson, Katherine L. Furniss, Carlos C. Goller, Jaime E. Mirowsky, Michael E. Moore, Samiksha A. Raut, Uma Swamy, Sue Wick, Michael J. Wolyniak

**Affiliations:** 1 Department of Microbiology and Immunology, Medical College of Wisconsin-Central Wisconsin, Wausau, WI United States; 2 Department of Health Sciences, Stetson University, DeLand, FL United States; 3 Department of Biology Teaching and Learning and Biotechnology Institute, University of Minnesota–Twin Cities, Minneapolis, MN United States; 4 Department of Biological Sciences, North Carolina State University, Raleigh, NC United States; 5 Department of Chemistry, State University of New York College of Environmental Science and Forestry, Syracuse, NY United States; 6 STEM Education Center, University of Arkansas at Little Rock, Little Rock, AR United States; 7 Department of Biology, University of Alabama at Birmingham, Birmingham, AL United States; 8 Department of Chemistry and Biochemistry, Florida International University, Miami, FL United States; 9 Department of Biology Teaching and Learning, University of Minnesota–Twin Cities, Minneapolis, MN United States; 10 Department of Biology, Hampden-Sydney College, Hampden-Sydney, VA United States

**Keywords:** ADKAR, Vision and Change, Active learning, Community of transformation, Mentorship, Professional societies, Professional development

## Abstract

One promising practice for increasing active learning in undergraduate science education is the use of a mentoring network. The Promoting Active Learning and Mentoring (PALM) Network was launched with practitioners from several professional societies and disciplines to make changes in their teaching based on evidence-based practices and to encourage the members to reflect deeply on their teaching experiences. Members of the Network interviewed seven previous Fellows, 1 to 6 years after completing their fellowship, to better understand the value of the Network and how these interactions impacted their ability to sustain change toward more active teaching practices. The interviews resulted in the creation of three personas that reflect the kinds of educators who engaged with the Network: Neil the Novice, Issa the Isolated, and Etta the Expert. Key themes emerged from the interviews about how interactions with the PALM Network sustained change toward evidence-based teaching practices allowing the members to readily adapt to the online learning environment during the COVID-19 pandemic. Understanding how the personas intersect with the ADKAR model contributes to a better understanding of how mentoring networks facilitate transformative change toward active learning and can inform additional professional development programs.

**Supplementary Information:**

The online version contains supplementary material available at 10.1007/s44217-022-00023-w.

## Introduction

The *Vision and Change* report led to a movement in undergraduate life science education to increase the centrality of evidence-based practices, including the incorporation of student-centered active learning approaches [1]. Active learning practices, when implemented well, create a learning environment in which all students are motivated to learn and have a high likelihood of passing the class [2–4]. High-impact practices, such as those that utilize collaborative in-class problem solving, encourage students to major in Science, Technology, Engineering, and Math (STEM) disciplines, improve scientific literacy, and promote students’ pursuit of STEM-based careers [5–7]. There is evidence for a positive impact of the *Vision and Change* movement*,* but questions remain about how to implement active learning practices and whether instructors who implement these practices sustain them, and if so, why.

Instructors are critical change agents, but often encounter barriers in incorporating evidence-based teaching strategies [8–12]. It is important to understanding what enables educators to overcome such barriers leading to sustained adoption of active teaching practices. The Prosci’s ADKAR model of change management can assist in understanding factors that support successful change. The ADKAR model focuses on guiding change at the individual level and includes an Awareness of the need for change, Desire to take part in the change, Knowledge of how to change, Ability to implement desired behaviors, and Reinforcement to sustain change [13]. (Throughout this paper we will use capital letters for Awareness, etc. whenever referring to interviewee comments and our conclusions that relate to the elements of the ADKAR model.) Some instructors are unaware of effective teaching practices, particularly if they trained in environments dependent primarily on didactic lectures. Even instructors with an Awareness of the need for change may not Desire to make change due to a perceived lack of time or institutional support [14]. Many science training programs do not provide students with the Knowledge to successfully implement active learning. In addition, not all institutions have resources like centers for teaching and learning to support their instructors’ Ability to implement active learning-based pedagogy. Sustained change comes from continued opportunities to Reinforce the desired behaviors, which can vary depending on the institutional commitment to and recognition of evidence-based efforts [14–16].

Several groups have used professional development workshops to promote active learning reforms in the undergraduate science classroom, including the Partnership for Undergraduate Life Sciences Education [17] (PULSE, *Partnership for Undergraduate Life Sciences Education—Empowering Departments, Transforming Education*, n.d.), the Summer Institutes on Scientific Teaching [18], the Center for the Integration of Research, Teaching, and Learning ([29]; *CIRTL Network Commons*, n.d.), and the American Chemical Society’s Collaborative New Faculty Workshop [19, 20]. However, evidence suggests that a workshop experience involving the development of active learning materials does not necessarily lead to a sustained impact on an instructor’s teaching practices [21–23]. Another strategy is through a community of practice that facilitates learning from experts while building supportive relationships with peers [24–27]. Communities of practice that support deep reflection about how science is taught and a commitment toward a new future are called communities of transformation (CoT, [28, 29]. A CoT strives to create an inclusive environment that brings people with different perspectives together around a shared commitment for substantive change [30]. Instructors in the CoT engage in multiple conversations on the application of teaching theories while iteratively improving their teaching practices.

The Promoting Active Learning and Mentoring (PALM) Network [15], funded by the National Science Foundation, and its precursor, the Mentoring in Active Learning and Teaching (MALT) program [31], funded by the American Society for Cell Biology, have used mentoring and the CoT approach to enhance the implementation of active learning in lecture-based courses. Each PALM Fellow identifies a teaching mentor with whom they interact individually, and the Network is formed by gathering Fellows, mentors, and steering committee members at conferences and in virtual spaces to discuss active learning. The steering committee includes staff members focused on education from a variety of life science professional societies.

Given a CoT approach is a promising practice, we wanted to better define what aspects of the Network can best support change for diverse groups of instructors. We used a phenomenological approach [32] combined with persona creation to describe why different instructors chose to participate in the PALM Network and the similarities and differences among these personas relative to the ADKAR change model.

## Methods

### PALM network structure

PALM Fellows work with a mentor and the CoT to implement active learning into their classroom through a structured, funded experience [15]. The PALM Network recruits applicants, including chemists and biologists from a variety of sub-disciplines, through a broad network of professional societies. The program director suggests mentors for about half of the applicants, while others find mentors through their own connections. Approval of mentors depends not only on the extent of their use of active learning in their own classrooms but also their record of promoting active learning through previous mentoring of colleagues, research publications, conference presentations, and leadership of workshops in active learning. Prospective Fellow-mentor pairs develop a project incorporating active learning and submit an application for review by two PALM steering committee members to evaluate the project design, experience of the mentor, and budget requests. The program has had 62 Fellows who meet with their mentor over at least one academic term. Fellows receive funds to travel to the mentor’s institution to observe their teaching and to reflect on how the mentor and sometimes the mentor’s colleagues employ active learning. Fellows and mentors also receive travel funds to attend a conference at which they present their PALM work. During and after the fellowship Fellows and mentors are encouraged to interact and co-mentor within the larger PALM Network’s CoT through journal clubs, bi-annual gatherings during professional society meetings, collaborative workshops/seminars, PALM-sponsored symposia, and additional projects aligned with the Network’s goals. Several Fellows and mentors whose formal PALM project ended years ago still participate in these CoT activities.

### Interviews

The research team, composed of individuals who have been PALM Fellows, mentors, and Steering Committee members, conducted semi-structured interviews in accordance with UMN IRB #1504E69564. We piloted and refined the interview script (Additional Information) to facilitate open-ended discussion. Interview participants included Fellows who had completed their formal mentorship 1 to 6 years prior to the start of our research project and who were not part of the study team (which had several members who otherwise were eligible for participation). The PALM Network program includes use of the Classroom Observation Protocol for Undergraduate STEM (COPUS, [33]) as one means of assessing a Fellow’s progress in incorporating active learning into their teaching. Time constraints, both for PALM Fellows and for COPUS coders, prevented us from a more intensive investigation of multiple pre- and post-mentoring videos, thus the COPUS data leaves us with only a snapshot of Fellows’ classroom activities. While acknowledging these limitations, we chose to use the existence of a Fellow’s pre- and post-mentoring COPUS data as one of the criteria for selecting interviewees since we believed that having Fellows review their COPUS data would encourage reflection on changes in their teaching.

Of the 12 Fellows who met the criteria for inclusion in our study, seven were available for interviews. The participants (five women/two men) included three from community colleges, one from a private liberal arts college, one from a 4 year public technical institute, one from a private R1 university, and one from an R2 public university (Additional file 1: Table S1). Pairs of authors conducted and recorded the interviews in Zoom (https://zoom.us). The interviews lasted approximately 1 hour and were transcribed by an independent transcription service and edited for accuracy.

### Coding and persona development

Initial coding was conducted by two researchers (A.P. and M.M.) to identify codes, each of which summarized a salient attribute for a portion of the data [34]. Six of the additional authors (S.W., K.F., C.G., J.M., H.E, U.S.) reviewed 2–3 transcripts to refine these codes. The group met several times to resolve any discrepancies and fine-tune the interpretation of the interviews, incorporating relevant insights based on their interactions with the Network. We found many of the codes overlapped with the ADKAR change framework and used Awareness, Desire, Knowledge, Ability, and Reinforcement as deductive themes to which we mapped our final inductive codes. The codes were organized in Excel and coding summaries were collected for each participant ensuring that claims were supported by participant quotes.

Persona Development is an approach that utilizes the primary information collected during interviews to identify key features and compiles the data in the form of fictional characters that are composites of multiple interviews [35, 36]. These rich, memorable descriptions incorporate users’ operational needs and individual preferences and can be useful to others developing similar professional development programs [37]. “User” is a general catch-all term that is technology-centric and the development of personas leads to a deeper human-level understanding of how individuals engage with an experience or product [35].

**Fig. 1:**
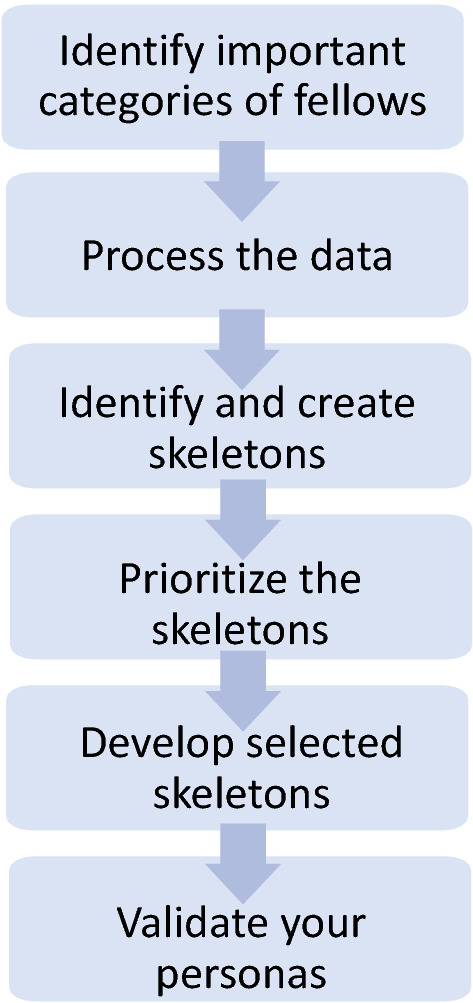
Overview of persona development. Six steps in the development of the personas. (Modified from [35])

There are 6 steps in the development of personas, including conception (steps 1–3) and gestation (steps 4–6) (Fig. 1). *Step 1: Discuss the categories of users* We determined that motivation and previous experiences were the most important aspect of the user experience with the PALM Network. *Step 2: Process the data.* Codes collected from the interviews were used to better understand the Desires and Knowledge of the instructors. *Step 3: Identify and Create Skeletons.* Our initial characterization led to the development of four skeletons based on the Fellows’ previous experience and familiarity with the theory behind active learning. *Step 4: Evaluate and prioritize skeletons.* We decided to consolidate down to three skeletons excluding the skeleton of an individual familiar with extensive theory but with little experience as there were limited data to support the inclusion of this skeleton. *Step 5: Develop skeletons into personas.* We noted key traits for each of the personas and the defining trait was identified to be used as part of the persona name. We randomly assigned gender utilizing Pruitt and Adlin’s recommendation [35] to employ alliteration for persona names to increase memorability. *Step 6: Validate personas.* The personas were validated amongst the group of authors to ensure that the personas reflect the whole data set, with every interviewee primarily represented by one persona while having a reflection in at least one other persona.

## Results

The PALM Network uses mentorship and engagement within a CoT to support the implementation of active learning in the undergraduate classroom with an ultimate goal of long-term, sustainable change in undergraduate science education. Previous Fellows were interviewed to identify their motivation for participating in the Network (which includes the Awareness and Desire elements of ADKAR), their experience implementing active teaching methods (Knowledge and Ability), and their teaching practices since participating in the fellowship, which may reflect Reinforcement.

**Fig. 2:**
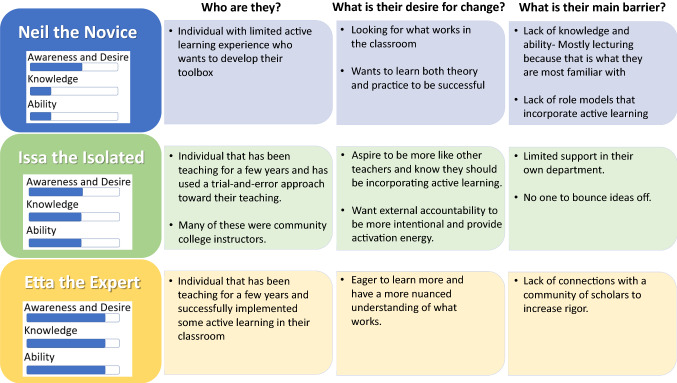
Summary of personas developed from interviews. The three personas that characterized the Fellows in the PALM Network were Neil the Novice, Issa the Isolated, and Etta the Expert. Each persona has a range of ADKAR features that represent their pedagogical development

The interviews led to the creation of three distinct personas within the PALM network (Fig. 2). The first persona, Neil the Novice, is excited to gain experience and Knowledge in active learning and to build his teaching toolbox since he has focused primarily on lecturing. Neil wants to understand the evidence for active learning and gain confidence implementing active learning in the classroom. Prior to his experience with the Network, Neil has had limited role models for employing evidence-based active learning. One of the Fellows, who was a postdoctoral fellow, stated:

I think what I felt was a disconnect, maybe an insecurity about my ability to apply these things in a real classroom with real students. And so I think having someone who had the experience to say, ‘This is great, but in reality, her’s how it might happen. And that's not a failure. It’s just going to look different than what you might theorize, or here are some roadblocks.

The second persona, Issa the Isolated, has tried some active learning, but has few colleagues with whom to discuss strategies and is not expected by her home institution to utilize active learning. She has tried a few strategies but aspires to use more active learning in her classroom. She wants the support of a community of like-minded people interested in active learning and Desires to have external accountability and Reinforcement to be more intentional. One of the Fellows shared:

I’ve been thinking about it for a long time. I just, again, didn’t have the support. I didn’t have a colleague that would help me. I didn’t have a Network like this, the PALM, that would be encouraging to me and to some extent, almost hold me accountable for things like this…In terms of active learning, I literally had no one to talk to, because no one was doing the things that I was doing.

The third persona is Etta the Expert. Individuals with this persona had been teaching for more than 5 years and had successfully incorporated active learning in the classroom. Etta is convinced that active learning is the way to teach and is a lifelong learner. She wants to try new techniques while increasing the rigor of her approach, recognizing that this will require additional Knowledge and Ability. Etta sees learning to teach as a career-long, iterative process that requires years to become an expert and one that benefits from continued scholarly collaboration with other educators. One of the more senior Fellows shared:

So I think it’s a never ending process. You have to keep learning and to become a better teacher. Because it doesn’t happen in 1 year or 2 years. It takes decades to become a master and if you want your students to be... Have a mastery towards their subject, have an expert-like thinking, then you have to become an expert in delivery of the content, which might take many years.

The interviews also helped us to better understand how the Fellows experiences led to sustained change.

The relevant codes mapped to the ADKAR framework and are included in Table 1.

**Table 1.**
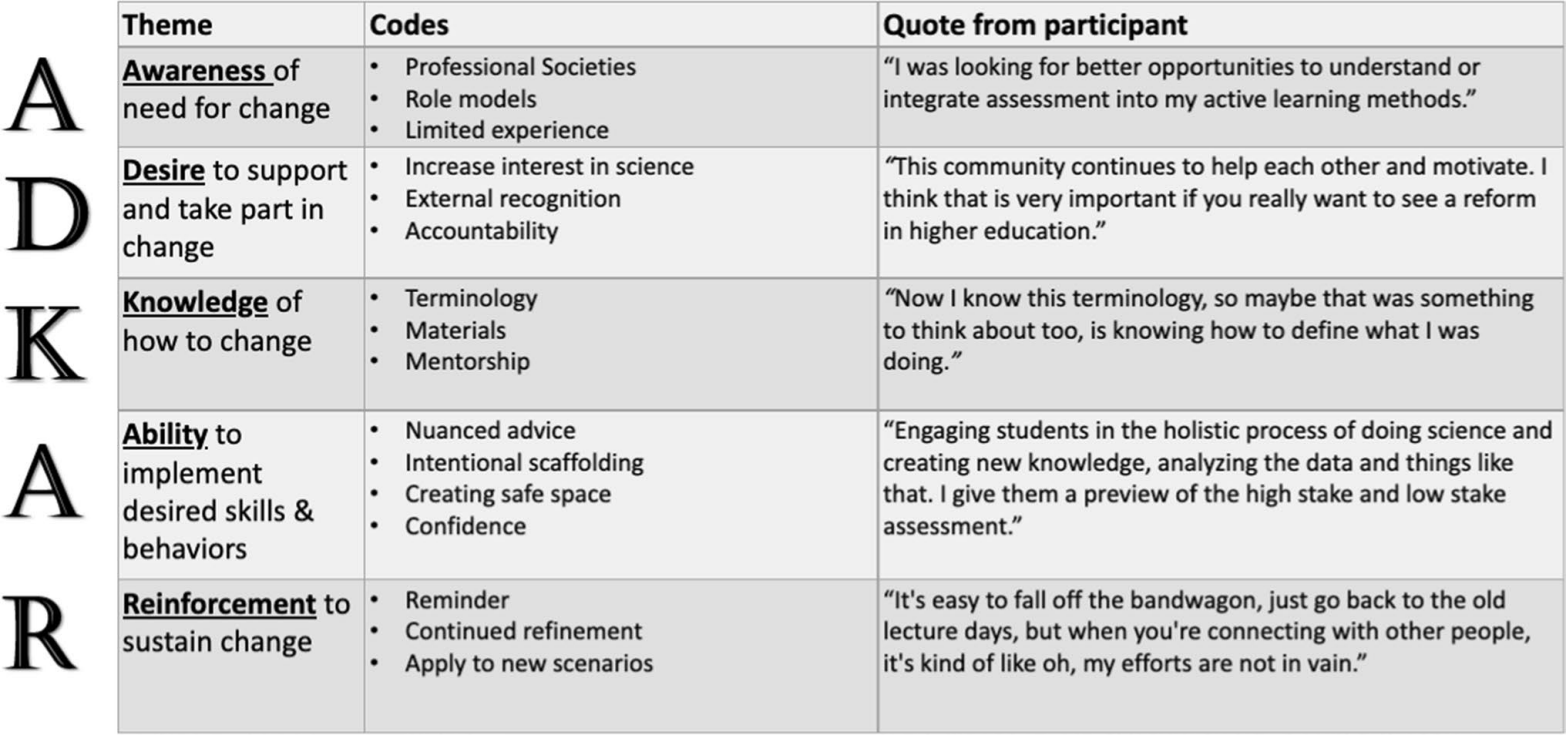
Alignment of codes and the ADKAR change model

### Awareness of need for change

By choosing to apply to the PALM Network, all personas were Aware of the need for change. They were aware of the program through professional societies and often identified role models whose teaching practices they emulated. Neil had a limited understanding of theory or best practices but described his students as “being bored” and unstimulated when giving lectures. Issa described using a narrow set of active learning approaches like clickers or think-pair-share exercises [38, 39]. One Fellow shared:

So prior to joining PALM, I was aware of active learning and I was doing some implementation of those tools... But my time allocated for active learning was very minimal, once a week or maybe twice a week

Etta had prior training in several techniques but is looking for better ways to fill in her own gaps for example by “integrating assessment into my active learning methods”.

### Desire to support and take part in the change

Fellows expressed a Desire to engage students in authentic scientific practices like collecting and analyzing data to assess student interest in science. One Fellow indicated:

Yeah, the grades are great. The cognitive stuff is great, but if we can change student attitudes and somehow measure that there’s a change in interest in students in science, wouldn’t that be nice?

The Fellows see being part of the Network as a chance to learn and grow, but also to gain external recognition and support. One Fellow indicated that financial “incentives help you, but I was interested in doing it anyway”. Issa the Isolated indicated that having the chance to interact with PALM members on a semi-regular basis held her accountable and provided the “needed activation energy” to make change.

### Knowledge of how to change

Another aspect of change is an increased Knowledge of how to make change. Neil the Novice gained the basic terminology to define what he was doing and a better understanding of the process of learning. One Fellow shared:

And so, I was able to spend a lot of time in the literature learning about how students learn, to tuck that into the revisions that I wanted to do and really be intentional about things that have been shown to improve people’s success in science.

Interacting with mentors allowed for brainstorming as well as sharing of materials. Issa the Isolated lacked colleagues to discuss teaching strategies within their home department and sought mentors with experience in the classes they teach. One Fellow said:

So what’s been helpful is by seeking out someone who had experience teaching the same kinds of classes I've been teaching, I could get actual handouts and protocols and instructions and things that have gone wrong when they had taught in the past.

### Ability to implement desired skills and behaviors

Fellows indicated that their mentors increased their Ability to implement active learning by giving them “nuanced advice” to achieve pedagogical goals. One Fellow shared:

So trying to do active learning and group work in a way that, I don’t know, that truly enriches understanding, as opposed to, “I’m doing some active learning so that I can say I’m doing active learning.” And so for the students, it’s not just about like variety, right? It’s hopefully serving a good pedagogical purpose.

The Fellows gained insight into how to design inclusive classrooms that engage all students by scaffolding, modeling, and assigning group roles. Additional techniques to support active learning included setting expectations upfront, explaining the “why” for engaging in group discussions, and giving students previews of assessments. Fellows described structurally reorganizing their classrooms to promote discussions and using the feedback from the students to help drive the flow of the session. One participant indicated:

There’s a small wheelhouse that you can go through, but it’s how you apply them and when you apply them and then, and how you manage it… You can have students do group work effectively or ineffectively, and it’s more in what makes something effective or ineffective. So I think understanding that distinction is what I was really looking for through my relationship with my mentor is like, I know about Think-Pair-Share, but when I might want to use it strategically, what I might be doing as students are doing Think-Pair-Share sort of those details in observing her class and then just talking about how things went, helps me be a lot more intentional.

Interviewees who fit the personas of Neil and Issa learned about the importance of building trust with the students and creating a safe space for the students to learn from their mistakes. A participant indicated “They (the students) are the ice skaters on the ice and are going to fall a lot”*.* Guidance from the larger Network provided the Fellows’ with the tools and confidence to support the students. One Fellow indicated.

There’s no way I would have had the confidence to completely burn down my lab and rebuild it with something completely different without the experience I had with my mentor. Without her saying, “It’s going to be scary and you’re going to be nervous and it’s going to be like you’re a new teacher again. And here’s how your administration is going to react. And here’s how your students are going to react.” Without that, that mentor experience with her giving me the confidence to do this, I don’t think I ever would have gotten over the hump to have the really positive experiences my students are having now. So, I think this is fundamental in being able to make that shift, which now will impact students for many years to come

### Reinforcement to sustain change

Etta the Expert recognizes the need for a continued commitment to active learning. One Fellow indicated, “I can’t imagine running a class without many components of active learning in it. I can’t do it and I won’t do it. I really feel the urge to continue to do what I’m doing*.*” The Fellows left the mentoring experience with a commitment to continue to adapt their teaching and seek out opportunities for Reinforcement both inside and outside of the Network to keep the momentum going. One participant indicated:

I think that’s the biggest thing that I will continue to evolve with after, and just continuing to read the literature and refining in every way that I can… It’s easy to fall off the bandwagon, just go back to the old lecture days, but when you’re connecting with other people, it’s kind of like oh, my efforts are not in vain. I'm not just randomly coming up with stuff. There’s people across the country who are doing things like that, and it helps for sure, so it’s encouraging to think of it that way.

The value of engaging in a journal club is described below by one of the Fellows:

Just reading those little journals, even though I’m not going to lie. I didn’t read every word of them but skimming those a little bit and then engaging and talking about them. I would spot the main ideas and I would refine my teaching and my mentality and mindset, mostly, based on that constant reminder. Even when active learning got hard, it was refreshing and kind of encouraging to have that narrative and to talk outside of my school, again, with like-minded individuals that have a similar background

Fellows shared how the Knowledge and Ability they gained from the fellowship was Reinforced in their successful pivot to online learning during the COVID-19 pandemic. An example provided by a Fellow:

When I would send them in the Zoom breakout rooms, I would have to design what I was asking them to do to try to recreate that process. Because I rely so much on moving between groups when sending them out or in Think-Pair-Share, just watching them and hearing the noise level of the classroom. So it was asking myself if I can’t listen to the groups talking, how else can I do it? And it was using Google slides and they had to fill in things. So I can at least see that they've all logged on, they’re using the slides and then like writing words in and I can see what they’re writing and if they’re on track or not. And I'm not sure I would have been able to do that as effectively had I not, both had experience of using group work, but also had the theory of understanding what it is we’re trying to accomplish in that group work. Because then with the theory it looked completely different, but at least I was accomplishing those same goals.

These comments show that the PALM Fellows who were interviewed are sustaining long-term pedagogical changes applying these principles to new learning environments.

## Discussion

Our study supports the benefits of using a Network to achieve a meaningful shift toward evidence-based teaching [27, 28, 40]. The interviews led to a richer understanding of the impact of the program on Fellows’ teaching and their long-term commitment toward shifting their teaching practices. One limitation of our results is that not all invited Fellows were available or chose to participate in the interviews, so our data may be skewed toward Fellows who have been more successful at implementing and sustaining active learning.

PALM Fellows recognized the benefits of having access to role models from outside their institutions. Mentorship led to an enhanced understanding of the need to support deeper learning and metacognition by helping students to plan and monitor their learning [41, 42]. If instructors utilize active learning techniques like clickers but fail to use these approaches formatively to direct student learning, they may not garner optimal benefits. Our interviews indicate that PALM Fellows are not just employing more active learning, they are doing it in a thoughtful, student-centered way, guiding their students to become more successful learners, and becoming more reflective and agile practitioners in both the classroom and in remote learning environments [43].

The personas we created clarify the similarities and differences among the Fellows. The use of personas is limited in professional development but has value in centering the potentially diverse needs of the end user [35]. Others have suggested that differing personas may require unique professional development [37]. Our results support that all personas benefited from their engagement with the PALM Network, but in different ways. The Network provided Fellows like Neil the Novice the Knowledge and Ability to create robust classroom discussions that support collaborative practices amongst students. Issa the Isolated was able to receive the critical peer support needed to adopt these practices. The long-term nature of the Network created multiple opportunities for Reinforcement, expanded the Fellows’ pedagogical perspectives, and led to the scholarly collaborative efforts that Etta the Expert sought.

## Conclusions

We found the ADKAR change model to be useful in determining factors leading to long-term change and suggest that it may assist others trying to promote and monitor change in higher education. Likewise, considering the development and use of personas may help to keep the intended change participants at the center of program design in order to better support their professional goals. Our interviews provide evidence of the value of engagement within a large, diverse network of instructors at multiple points in their careers. Teaching reform programs generally promote increased Awareness, Desire, and Knowledge elements of the ADKAR model. The Network approach additionally engages Fellows in continued reflection with their mentors increasing their Ability to successfully implement these changes. We find that mentoring partnerships can initiate change, and that linking of partnerships through shared activities like leading workshops, writing publications and grant proposals, and journal club discussions generates a Network of interactions that Reinforces long-term, sustainable change.

## Supplementary Information

Below is the link to the electronic supplementary material.

**Additional file 1: Interview Script Table S1.** Table of Participants

## Data Availability

The datasets generated during and/or analyzed during the current study are available from the corresponding author on reasonable request.
