# Supplementary material for: Using personas and the ADKAR framework to evaluate a network designed to facilitate sustained change toward active learning in the undergraduate classroom
Source: Discov Educ. 2022 Dec 27;1(1):22. doi: 10.1007/s44217-022-00023-w (PMC9793354; doi:10.1007/s44217-022-00023-w)
Supplement: Supplementary file 1 — Additional file 1: Interview Script Table S1. Table of Participants [file 44217_2022_23_MOESM1_ESM.docx]

**Additional file**

**Interview Script:**

What prompted you to apply for a PALM Fellowship?  What were you looking for, both in a narrow techniques sense and in a broader pedagogical/teaching philosophy sense?

To what extent were you looking for (or, did you find) a community of practice above and beyond the individual interaction with your mentor?

Describe what type(s) of active learning technique(s) you used during your fellowship and what changes you tried to make.

What are the thought processes you go through when deciding to design, implement, and assess active learning elements of your courses?

What kinds of changes do you see in your students as a result of your efforts to expand your use of active learning? This could take the form of qualitative impression, attitudinal changes of students in the class, or performance in the class.

Do you notice any attitudinal changes to yourself as an instructor?

What is the story behind your COPUS results?

Describe how your teaching has evolved as a result of the fellowship.

Table S1. Table of Participants

| Interviewee | Position | Institution type | Years between start of fellowship and interview |
| --- | --- | --- | --- |
| A | Postdoc | R1/medical school | 5 |
| B | Instructor | Community college | 2.5 |
| C | Assistant professor | Liberal arts college | 6 |
| D | Tenured instructor | Community college, others as adjunct | 3.5 |
| E | Instructor and PhD student | R2 research university | 1 |
| F | Associate professor | Technical institute | 1.5 |
| G | Adjunct professor | MSI community college | 4.5 |
